# Central nervous system manganese induced lesions and clinical consequences in patients with hereditary hemorrhagic telangiectasia

**DOI:** 10.1186/s13023-017-0632-2

**Published:** 2017-05-18

**Authors:** M. M. Serra, C. H. Besada, A. Cabana Cal, A. Saenz, C. V. Stefani, D. Bauso, A. B. Golimstok, J. C. Bandi, D. H. Giunta, C. M. Elizondo

**Affiliations:** 1 Internal Medicine Department. Hospital Italiano de Buenos Aires. Argentina (HIBA), Presidente Perón 4190, Cuidad Autónoma de Buenos Aires, C1199ABB Argentina; 2 Radiology Department, HIBA, Ciudad Autonoma de Buenos Aires, Argentina; 3 HHT Unit. Hospital Italiano de Buenos Aires, Ciudad Autonoma de Buenos Aires, Argentina; 4 Neurology Department, HIBA, Ciudad Autonoma de Buenos Aires, Argentina; 5 ARG (Argentine Rendu Study Group), Ciudad Autonoma de Buenos Aires, Argentina; 6 Internal Medicine Research Unit, HIBA, Ciudad Autonoma de Buenos Aires, Argentina; 7 Hepatology area, HIBA, Ciudad Autonoma de Buenos Aires, Argentina

**Keywords:** Hepatic vascular malformations, Hereditary hemorrhagic telangiectasia, Basal ganglia manganese deposits, Iron deficiency anemia

## Abstract

**Background:**

Around 47–74% of patients with hereditary hemorrhagic telangiectasia (HHT) have hepatic vascular malformations (HVMs); magnetic resonance images (MRI) of the central nervous system (CNS) might show in T1 sequences a hyper-intensity signal in different areas, mainly in the basal ganglia (BG) as consequence of manganese (Mn) deposits as observed in cirrhotic patients. These patients might suffer from different neuropsychiatric disorders (hepatic encephalopathy). In HHT patients, even in the presence of hepatic shunts, hepatocellular function is usually preserved. Additionally, Mn shares iron absorption mechanisms, transferrin and CNS transferrin receptors. In iron deficiency conditions, the Mn may harbor transferrin and access BG. The objectives were to describe frequency of BG Mn deposit-induced lesions (BGMnIL) in HHT patients, its relationship with iron deficiency anemia (IDA) and HVMs. Finally, explore the association between neuropsychological and motor consequences. We performed a cross-sectional study. We determined HHT patients with or without BG-MnIL by the MRI screening of the CNS. We included all patients with lesions and a random sample of those without lesions. All patients underwent standardized and validated neuropsychological assessment to evaluate BG actions. Results were analyzed with multiple logistic regression, adjusting for potential confounders.

**Results:**

Among 307 participants from a cohort included in the Institutional HHT Registry, 179 patients had MRI performed and Curaçao Criteria ≥3. The prevalence of BG-MnIL was 34.6% (95%CI 27.69-42.09). While neuropsychological symptoms were present in all patients, BG-MnIL patients performed poorly in three of the neuropsychological tests (serial dotting, line tracing time, number connection test A). HVMs frequency in BG-MnIL was 95.1%, versus 71.4% in those without lesions (*p* < 0.001). IDA frequency was 90.3% versus 54% (*p* < 0.001). When IDA is present, estimated risk for BG-MnIL is remarkably high (OR 7.73, 95%CI 2.23–26.73). After adjustment for possible confounders (gender, age, presence of HVMs), IDA was still associated with increased risk of BG-MnIL (adjusted OR 6.32, 95% CI 2.32–17.20; *p* < 0.001).

**Conclusions:**

Physicians should assess BG-MnIL in HHT patients in CNS-MRI. IDA and HVMs present increased risk of lesions. Patients with BG-MnIL have neuropsychological impairment, and they might benefit from sparing IDA, or undergoing future therapeutic options.

**Trial registration:**

NCT01761981. Registered January 3^rd^ 2013.

**Electronic supplementary material:**

The online version of this article (doi:10.1186/s13023-017-0632-2) contains supplementary material, which is available to authorized users.

## Background

The hereditary hemorrhagic telangiectasia (HHT) or Rendu Osler-Weber syndrome is an autosomal dominant inherited disorder characterized by an abnormal development and remodeling of the blood vessels. It affects around 1-5000/8000 individuals worldwide [1–6]. Clinical diagnosis of HHT is based on Curaçao criteria (spontaneous recurrent epistaxis, telangiectases in typical locations, visceral arterio-venous malformations -lung, liver, brain, and central nervous system- and first-degree family member with HHT) and/or genetic testing [3, 7].

Hepatic vascular malformations (HVMs) may be present in 41–74% of cases according to the screening method used and genotype [8]. The HVMs are more frequently found in patients with HHT2 [8–10]. Only 8% of patients have symptoms related to HVMs such as high output cardiac failure with secondary pulmonary hypertension, portosystemic encephalopathy, portal hypertension syndrome, steal syndrome or ischemic necrosis in the biliary tree. Symptoms related to HVMs are rare in patients before 50 years old [11]. In HHT patients, like cirrhotic patients and porto-systemic shunts, the central nervous system magnetic resonance images (MRI) in T1 sequences might show a hyper-intensity signal in different areas, mainly in the basal ganglia (BG) [12]. The patients with advanced hepatic disease who present Mn in their BG might suffer from different neuropsychiatric disorders known as hepatic encephalopathy or minimal hepatic encephalopathy [13, 14].

Studies in animals and humans have demonstrated that the T1 signal hyper-intensity found in different brain structures in patients suffering from liver diseases corresponds to manganese (Mn) deposits which are facilitated by hyper-ammonemia and/or hypermanganesemia [15]. On the other hand, the Mn uses the same absorption (Divalent Metal Transporter 1-DMT1), transport and deposition mechanisms as iron. In turn, iron deficiency might lead to the increase of manganese absorption which is harbored by the transferrin. High levels of transferrin receptors may be found in many CNS areas, predominantly in BG [13, 15–17]. Even in the absence of hepatic disease, iron deficiency collaborates with the increase of Mn absorption and deposition in the BG leading to a toxic effect [13]. The frequent iron deficiency anemia due to nose and gastrointestinal bleeding in HHT patients plus the presence of HVMs may result in a proper scenario to facilitate the deposit of manganese in the CNS.

Nowadays, in HHT patients, the prevalence of deposits of Mn in the BG, their pathophysiologic mechanisms and clinical implications are not well established [18, 19]. The purpose of this study is to determine the prevalence of BG-MnIL and their risk factors in HHT population and to evaluate possible neuropsychological and motor consequences.

## Methods

We conducted a cross sectional study in a multidisciplinary HHT care unit located at the “Hospital Italiano de Buenos Aires”, a University hospital. The HHT Unit consists of different specialists majored on HHT who assist HHT patients from different countries of our region from the moment of diagnosis. There is no severity or manifestation-type referral bias, the patients are referred for clinical evaluation and treatment. All patients underwent standardized screening assessment recommended for HHT patients [3].

Study population was made up of adult patients with confirmed HHT (3 or more Curaçao clinical criteria and/or genetic test) who were included in our Institutional Registry of HHT -clinicaltrials.gov NCT01761981- and had a brain MRI screening performed. We excluded those who refused to the informed consent. Other exclusion criteria were neurologic or psychiatric diseases, psychiatric treatment, alcoholism, hepatic diseases different from HHT HVMs and portal hypertension. The protocol was approved by our Institutional Review Board. The study was performed from June 2012 to December 2014.

### Study procedures

The patients included in the Institutional registry of HHT who met the inclusion criteria for this study, were contacted by phone and/or e-mail for an interview with the principal investigator inviting them to participate in the study. In the interview, study purpose and procedures were explained in detail, and patients were requested for the informed consent. Patients were included prospectively. Once included, MRI images were send to a neuro-radiologist, laboratories and hepatic studies were registered and a random sample of those presenting BG-MnIL and those without BG-MnIL were derived for neuropsychological evaluation.

All screening MRI of the CNS from patients included in this study underwent exhaustive revision of their images focusing on BG and other locations with hyper-intensity images consistent with Mn deposits and vascular malformations. Prevalent cases were defined in those patients in which BG-MnIL were detected in the screening MRI. The MRIs were performed with RM equipment 1.5 T with and without gadolinium sequences. Axial and sagittal images in T1 and T2 diffusion sequences were performed as standard. Data processing was blind to patient clinical information in a Siemens commercial work station (Syngo). The MRI images were analyzed by two independent high trained neurorradiologyst through visual inspection in all cases. They define BG-MnIL with hyperintensity images in T1 in the BG area.

A randomized sample of patients -simple random sampling method- with BG-MnIL and without BGMnIL underwent a detailed neurological examination by neurologists specialized in movement disorders to detect extrapyramidal signs and symptoms. All patients were assessed with a comprehensive neuropsychological battery conducted by neuropsychologists with experience in brain disease. The neuropsychological assessment protocol included the “Psychometric Hepatic Encephalopathy Score” (PHES), Fig. 6, see Additional file 1.

The PHES has been standardized in several countries, such as Germany, Italy, Spain, India, Korea and Mexico. The quantitative measurement of performance in each subtest of PHES and the tests, was judged as deficient, when the value was 1 SD or more below means score in standardized scale [20, 21]. As the Argentinian standardization of this battery is not finished, the results of the NCT-A, NCT-B, and SDT were measured in seconds, including the time needed to correct any errors, and the result of DST was measured in points. The results of the LTT were measured as both the time needed to complete the test (LTTt, seconds) and as the number of errors (LTTe) [20, 22, 23]. Poor outcomes are represented by prolonged times and greater number of errors.

To screen the global cognitive performance of patients we used the known Mini-Mental State Examination (MMSE) [24]. The MMSE or Folstein test is a 30-point questionnaire that is used extensively in clinical and research settings to measure cognitive impairment [25].

Hepatocellular function was assessed as standard in HHT patients’ medical care, with liver function test, prothrombin time or factor V dosage. The hepatic vascular malformations were identified by standardized Doppler ultrasound and/or enhanced triphasic multislice computed tomography (MSCT) and classified by physicians with high training in HHT images [3, 26, 27]. We included in this study the worst values a patient could have regarding hepatic compromise in 1 year time from MRI assessment.

Anemia and iron deficiency state was evaluated, as usual blood tests are included in our HHT screening protocol: Hematocrit, hemoglobin, ferritin, iron serum levels and transferrin saturation. We define anemia with hemoglobin below 12,5 g/dl or 11,5 g/dl in males and females respectively. We considered iron deficiency anemia (IDA) when ferritin was below 20 ng/ml, when inflammatory conditions was ruled out, or when transferrin saturation was below 16% [28, 29]. We included in this study the worst values a patient could have in 1 year time from MRI assessment.

### Statistical analysis

Patients included in this study were considered the denominator for the BG-MnIL prevalence estimation. We reported prevalence with 95% confidence interval.

Comparisons between groups with BG-MnIL and without BG-MnIL were tested with chi square test and Man Whitney.

We estimated risk of presenting BG-MnIL in patients with IDA and reported Odds Ratios with 95% confidence intervals. Multivariate logistic regression analysis was performed to adjust for confounding variables (age, gender and HVMs).

To stablish BG-MnIL impact on neuropsychological symptoms we performed a linear regression model for each test and adjust for confounders (IDA and HVMs). We evaluated if the interaction between IDA and HVMs could affect neuropsychological symptoms when presenting BG-MnIL. We considered statistical significance p values <0.05. All analysis was performed with STATA software vs 14.

### Sample size

Manganese deposits prevalence was estimate in the total HHT cohort. For the objective of comparing the presence of neurological affectation in BG-MnIL patients, we calculated a sample size considering an estimated difference of 35% (40% of patients with BG-MnIL would have psychological symptoms and only 5% of those without lesions), power on 80% and alpha 0.05, we estimated 20 patients with BG-MnIL and 20 without it.

## Results

**Fig. 1:**
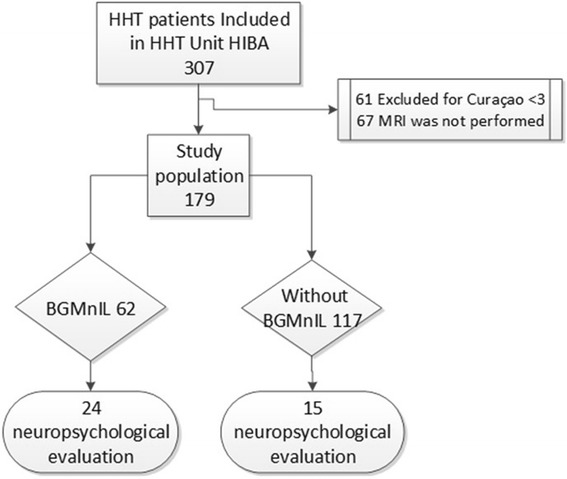
Flow chat of participants. There are two groups, 62 patients presented BG-MnIL and 117 patients without BG-MnIL. The figure shows patients who presented neuropsychological evaluation in each group

From a cohort of 307 patients included in the Institutional HHT registry, we included 179 patients who had MRI performed and Curaçao Criteria > = 3. See patient flow chart (Fig. 1).

The estimated BG-MnIL prevalence was 34.6% (95%CI 27.6%- 42% -62/179 patients) in HHT cohort.

We found a high prevalence in patients of female gender (69.3%) and young patients (median age 46 years, interquartile range 25). IDA and HVMs were more prevalent in HHT patients with BG-MnIL (Table 1).

**Table 1:** Basal characteristics

Characteristics	HHT patients (179)	BG-MnIL patients (62)	Patients without BG-MnIL (117)	*P* values
Female gender^a^	69.3% (124)	75.8% (47)	65.8% (77)	0.16
Age in years^b^	46 (25)	55.5 (20)	41 (25)	0.058
Iron Deficiency Anemia^a^	58.1% (104)	87.1% (54)	42.7% (50)	<0.001
Hepatic Vascular Malformations^a^	80% (128/159)	95.1% (58)	71.4% (70)	<0.001
Ferritin ng/ml^b^	14 (30.7)	14.1 (9.75)	22 (24.7)	0.004
Hemoglobin g/dl^b^	11.8 (3.8)	10 (7.7)	12.2 (3.1)	0.004
Hematocrit %^b^	36 (10)	30.9 (14)	37 (9.35)	0.004
TF Saturation %^b^	15% (17.6)	9.5 (12)	19 (15)	0.003

^a^% (absolute frequency), ^b^ medians (interquartile range). Reference values: Hematocrit: 37% in men and 36% in women, Hemoglobin 12.5 g/dl in men and 11.5 g/dl in women, Ferritin: 20 ng/ml, Transferrin Saturation (TF) >16%

**Fig. 2:**
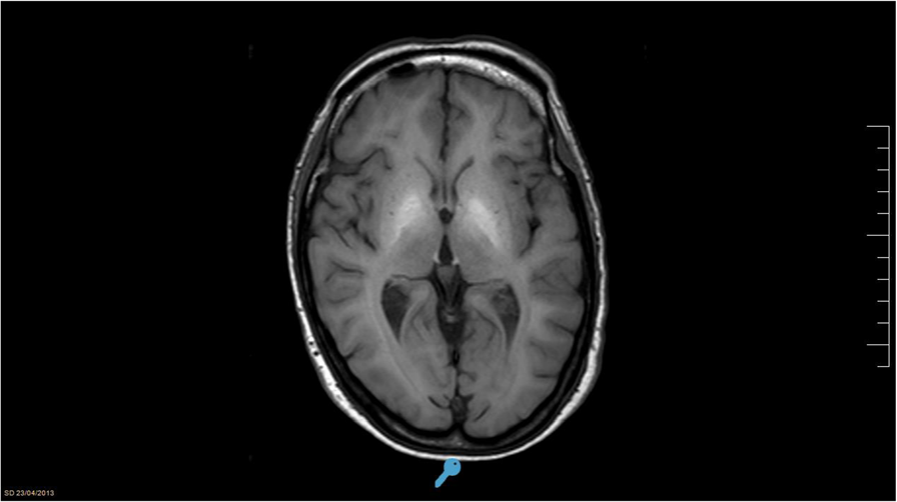
Psychometric Hepatic Encephalopathy Score. The figure shows a schematic representation of test included in PHES battery

**Fig. 3:**
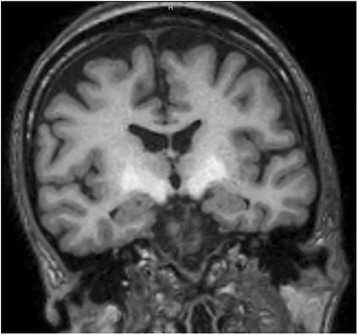
BG-MnIL MRI image. T1 sequence Brain MRI showing hyperintensity signal in the basal ganglia as consequence of manganese deposition

**Fig. 4:**
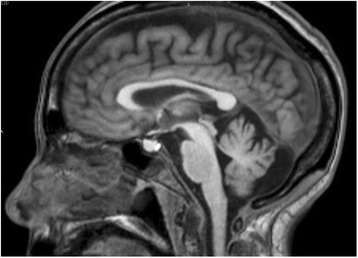
Manganese deposits in CNS structures. Coronal MRI T1 image showing hyperintensity signal in the midbrain as consequence of manganese deposition

The most frequent CNS structure affected was the globus pallidus (40 patients), other sites were hypophysis (11), midbrain (10) and others: putamen and caudate (1), periaqueductal grey matter (1), protuberance (1). Characteristic lesions are seen in brain MRI as shown in Figs. 2, 3 and 4.

**Fig. 5:**
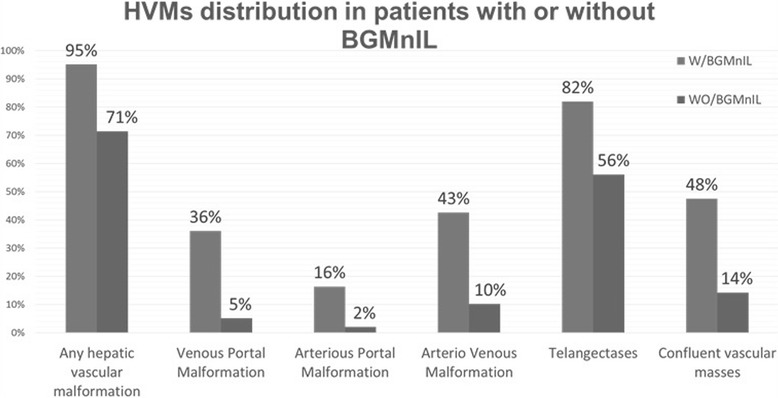
Manganese deposits in CNS structures. Sagittal MRI T1 image showing hyperintensity signal in the adenohypophysis as consequence of manganese deposition

All kind of HVMs were more frequent in the BG-MnIL group. The most frequent were the telangectases, even more so, in many patients the telangectases were the only kind of HVM detected (isolated telangectases in 7/62 patients with BG-MnIL and in 33/117 patients without them) (Fig. 5).

**Fig. 6:**
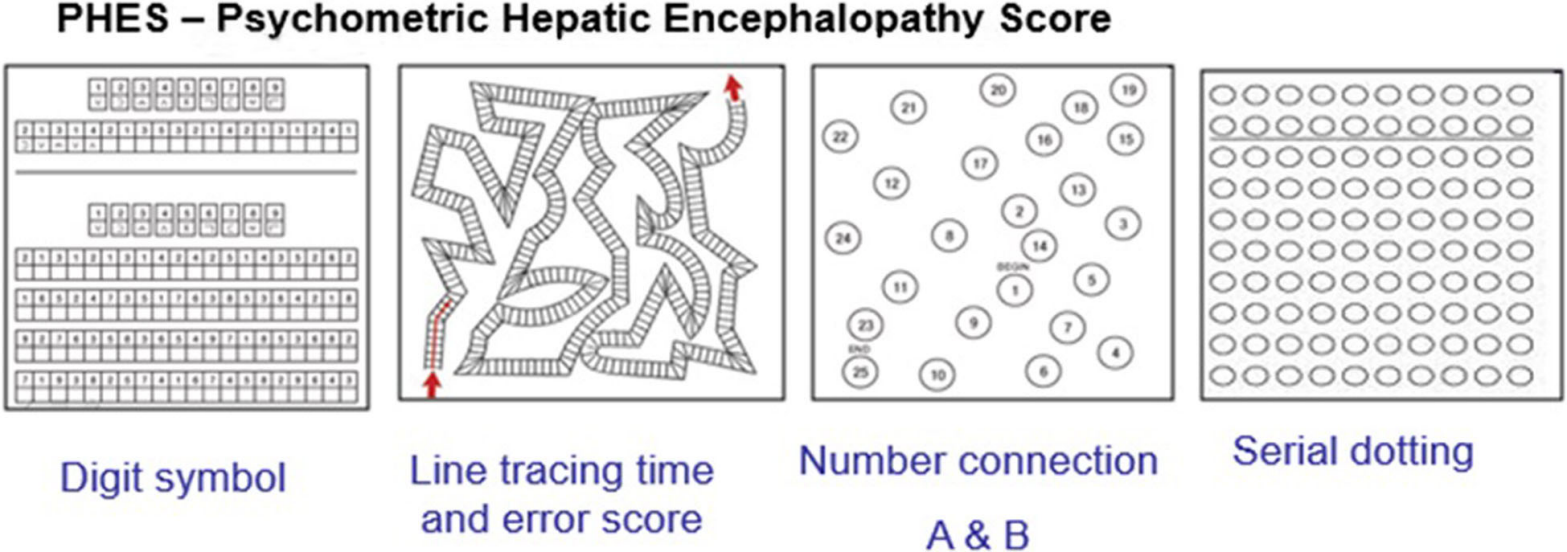
Hepatic vascular malformations in HHT patients. This figure shows the distribution of the different kind of hepatic vascular malformations in patients with BG-MnIL and without BG-MnIL

### Basal Ganglia Manganese Induced Lesions Risk

The risk of having BG-MnIL in IDA patients was estimated with a crude OR 7.95 (CI 95% 3.13–20.13) *p* < 0.001. The association between BG-MnIL and IDA adjusted by gender, age, presence of hepatic vascular malformations was OR 6.32 (CI 95% 2.32–17.20, *p* < 0.001).

The risk of having BG-MnIL in patients with HVM was estimated with a crude OR 7.73 (CI 95% 2.23–26.73) *p* < 0.001. The association between BG-MnIL and hepatic vascular malformations adjusted by IDA was OR 6.49 (CI95% 1.75–24.02) p 0.005, and adjusted by IDA, age and gender was OR 5.31 (CI95% 1.39–20.32) p 0.014.

#### Neuromotor assessment and Neuropsychological assessment results

Twenty-four patients with BG-MnIL and 15 patients without BG-MnIL underwent neurological motor assessment focused on abnormal movements. Eleven patients with BG-MnIL (47%) were affected in a neuromotor level. Patients without BG-MnIL did not present any motor neurological affection. The different clinical findings were tremors 7 (28%), rigidity/Parkinsonism 2 (16.5%), myoclonus 1 (4.7%), restless legs syndrome 1 (4.7%).

The same patients with BG-MnIL and without BG-MnIL underwent the neuropsychological assessment protocol. We found neuropsychological failures in both groups in tests that assessed speed and finely-tuned motor control when comparing to original standards. Nevertheless, the failures have been significantly greater in the group with BG-MnIL when considering times and numbers of errors. We also found failures in tests that assessed focus and concentration, in both groups. We have not found relevant failure in executive functions. We could not find cognitive features within patients who shared the Mn location. The only patient that passed all the tests had Mn only in adenohypophysis. Neither the age nor the education showed impact on the performance. In Table 2 we presented comparison of absolute values of time in seconds for Serial Dotting Test, Line Tracing Test-time, Number Connection Test A and B, number of errors in Line Tracing Test-errors and points in Digit Symbol Test. Worse time or numbers of errors are interpreted as poorer neuropsychological performance.

**Table 2:** Neuropsychological test values by group

Neuropsychological test	Patients with BG-MnIL (*n* 24)	Patients without BG-MnIL (*n* 15)	*p*
Serial Dotting Test -in seconds-	68 (57–85)	59 (54–62)	0.01
Line Tracing Test (time) -in seconds-	117 (86–147)	93 (80–121)	0.09
Line Tracing Test (errors) -number of errors-	9 (2.5–17.5)	6 (1–8)	0.08
Number Connection T A -in seconds-	40 (28–55)	33 (24–38)	0.03
Number Connection T B -in seconds-	82 (60–106)	69 (57–86)	0.11
Digit Symbol (points) -number of points-	43 (36–49)	47 (40–52)	0.37
MMSE -score number-	30 (29–30)	30 (29–30)	0.39

Numbers as median (25–75 percentiles)

Given that all patients presented at least one neuropsychological symptom was present in all patients, BG-MnIL, both with and without BG-MnIL, we estimated the BG-MnIL effect in a lineal regression for each neuropsychological test and estimated coefficients. We found statistical significance in Line Tracing Test -errors, Serial Dotting Test and Number Connection Test A. We performed an adjusted model by gender, age, IDA and HVMs and an interaction term between IDA and HVMs. After adjusting, the impact of having BG-MnIL is still significant for Number Connection Test A and Line Tracing Test- errors (Table 3).

**Table 3:** BG-MnIL impact on neurological symptoms. Lineal Regression for each test (crude and adjusted)

Neuropsychological test	Crude Coefficient (95%CI)	*P*	Adjusted Coefficient* (95%CI)	*p*
Serial Dotting Test	21.1 (2.3–40)	0.02	17.0 (−4.6–38.7)	0.12
Line Tracing Test (time)	31.4 (−4.7–67)	0.08	25.5 (−16.8–67.8)	0.22
Line Tracing Test (errors)	6.5 (0.1–13)	0.04	7.6 (0.1–15.1)	0.04
Number Connection T A	15.0 (2.7–27)	0.01	14.7 (0.3–29.1)	0.04
Number Connection T B	19.9 (−3.3–43)	0.09	20.5 (−7.0–48.0)	0.13
Digit Symbol (points)	−3.6 (−10–3)	0.28	−2.3 (−10.3–4.8)	0.47

Adjusted by gender age, IDA, HVMs and interaction IDA*HVMs

## Discussion

This study presented basal ganglia manganese-induced lesions prevalence in HHT, their risk factors and neurological impact. We found BG-MnIL in one third (34.6%) of patients with HHT mainly in the globus pallidus. IDA was identified as an independent risk factor for BG-MnIL. IDA is a frequent event in HHT being the main modifiable risk factor for manganese deposits. Thusly, the present study highlights a non-well known clinical feature secondary to IDA in HHT patients [1].

We also found that BG-MnIL might be responsible for neuropsychological and motor impairment that remains unnoticed for majority of patients in their daily activities. This scenario could suggest a greater risk of lifelong damage exposure. On the contrary, this would probably require prompt diagnostic and therapeutic actions.

Although there are few reports of small case series which describe hyperintense basal ganglia lesions in HHT population, none of them evaluated their neurological impact. Moreover, they only postulated HVM as their main risk factor leaving aside ferropenic states [12]. Ihara K et al. reported two non-HHT patients in whom portosystemic shunts were associated to BG-MnIL in the presence of Mn in workplace exposure [15]. K Yoshikawa et al. reported the first case suggesting IDA as a potential risk factor in a young woman with HHT, who presented parkinsonism, portosystemic shunts and elevated Mn blood levels who present BG-MnIL [14]. In this report they postulated physiopathology mechanisms for IDA involvement but the neuropsychological description is limited.

There are other studies that explained the relation between iron deficiency and BG-MnIL but in a non-HHT population Also manganese related encephalopathy is well descripted in cirrhotic patients, welders and patients receiving parental nutrition. Normally, the liver carries out the manganese clearance through the biliary excretion. In the case of hepatic disease as cirrhosis or portosystemic shunts, the manganese clearance may be decreased leading to high serum levels of this cation. The increase of ammonia in those patients may lead to an increase of glutamine and decrease of glutamate, myoinositol and choline in astroglia. In turn, these changes lead to an increase of vascular permeability facilitating the access and deposits of manganese in the CNS [30, 31]. Manganese produces a neuronal toxic effect leading to reversible or irreversible neuronal injury, depending on the duration of the manganese exposition [13]. It has also been associated to parkinsonism and psychometrics test deficit caused by dopaminergic neurotransmission impairment in basal ganglia [32]. According to Weissenborn there was no relation between MRI hyperintensity density and neuromotor or neuropsychological state in cirrhotic patients, though neurologic symptoms were present according to hepatic lesion severity [33].

Although in the present study we did not performed MRI measurements of BG-MnIL hyperintensity to establish association between neurological symptoms and intensity gradient, we found different neuropsychological impairment in those without lesion, supporting our hypothesis. Despite our population suffering from HHT, this study collaborates with the comprehension on the basal ganglia diseases and their clinical consequences, perhaps commonly with subclinical presentation.

We have also noticed that other CNS structures were harbouring Mn, and that the adenohypophysis seemed affected in many cases. However, further studies are needed to evaluate the association between adenohypophysis lesions and endocrine symptoms. Neuropsychological and motor failures shown in BG-MnIL patients correlate with motor and neuropsychological failures related with speed task execution, concentration, focus, finely-tuned motor control and extrapyramidal symptoms, in concordance to the globus pallidus affection, the most frequent location of Mn in the CNS in this study. While PHES is validated in many countries there is no Argentinian validation. There are available validations and standard scores for Italian, Spanish and Mexican population. As the Argentinian population might have similar educational level to those countries, we believe it might be an evaluation battery that could be comprehensive and properly used in our population. Still, as we lack of Argentinian validation and standard scores for our population, we compare unprocessed time values and number of errors. Worse results represent that the task slows down.

HHT hepatic disease is assessed through hemodynamic consequences like high output cardiac arrest, secondary pulmonary hypertension, and portal hypertension syndrome with or without encephalopathy or ischemic damage of the biliary tree due to different kinds of vascular malformations [3]. It has been reported that only around 8% of patients with HVMs have shown the symptoms mentioned above in which the neurological consequences are caused only by portosystemic shunts. Contrary to the general knowledge that hepatic telangectases do not represent a relevant clinical problem, we have found that only the presence of hepatic telangectases might be sufficient, in certain cases, to ease the manganese deposition in the CNS in presence of IDA. It becomes significantly important since IDA and hepatic telangectases are very frequent among HHT patients.

On the other hand, not all HHT patients with HVMs showed manganese in the BG, making this a necessary risk factor might not be a sufficient explanation. Therefore, we studied another mechanism to understand this phenomenon. As we mentioned, we found that the iron deficiency anemia is the main factor to ease the manganese deposits in the basal ganglia. In iron deficiency states, manganese harbours the DMT-1, transferrin and its receptors, which are mainly present with high density in the BG area, especially in the globus pallidus [14]. We have noticed that the IDA increases 6-fold the risk of Mn deposition in HHT patients regardless of the presence of HVMs, gender and age, being the most important risk factor we found in our research. Therefore, minimizing the IDA in the affected patients might reduce the magnitude of Mn deposits in the CNS improving the neuropsychological and neuromotor failures. Hence, iron deficiency and IDA might explain per se some psychomotor impairments, since the normal iron map distribution in the brain is the same as dopamine [34]. Moreover, the iron is a co-factor of catecholamine synthesis pathway. Is clear that iron deficiency and IDA cause cognitive, learning and behavioral impairments in children and perhaps in adults [35]. Some authors have found that iron supplementation in adults with IDA correlated with a better mental and speed task performance [36]. Nevertheless, in our study BG-MnIL is associated to neuropsychological symptoms independently from IDA and HVMs. Thus, further studies must face if these findings have some impact in everyday life of HHT patients because in our population none of our patient self-reported complaints. We could not assess time extent of iron deficiency anemia so we cannot demonstrate that being depleted of iron chronically could worsen manganese deposits or their neuropsychological impairment. We believe that the question that follow these results, first, is whether BG-MnIL is reversible, as happens in cirrhotic patients after liver transplantation and if their impact in neuropsychological tests will improve after IDA treatment.

Limitations.

The study design could not allow us to establish causality, nevertheless we highlight BG-MnIL and the association with iron deficiency anaemia should be consider in the HHT population.

There might be an overlap between minimal hepatic encephalopathy and symptoms associated with basal ganglia dysfunctions especially in patients with high portosystemic shunt. Nevertheless, in our study, only one patient presented evidence of hemodynamic compromise by portal hypertension.

The number of neuropsychological evaluations might be insufficient, all the same, we found some differences in this random selection of patients.

When we considered PHES standardized scores from other countries validations, all patients have at least one abnormal test in comparison to other standards. It would be necessary to validate neuropsychological tests in Argentinean population and contrasts our findings. Nevertheless, we believe that there have been no bias in the evaluation itself (specialized and standardized evaluation) or in population bias (random sample of cases and controls).

We decided to report test values demonstrating differences between groups. Although this is not the standard procedure, in some tests, values are sufficiently different in both groups, suggesting poorer outcomes in BG-MnIL. Prolonged execution times and number of errors might demonstrate enough neuropsychological lesion impact. Further studies will be required to determine long term impact of BG-MnIL.

We are concerned about the external validity of our findings, however, patients in this study are heterogeneous regarding their disease features, age, gender and socio-cultural or educational level, increasing the generalization of the results.

## Conclusion

We consider that patients with HHT should be assessed for BG-MnIL in their screening MRI, especially in those with HVMs and iron deficiency anemia. Probably it would be also useful to make a neuropsychological evaluation in those who presents lesions. There is still need to elucidate whether these lesions are reversible and if iron deficiency anemia treatment could contribute to ameliorate Manganese deposits.

## Additional file

Additional file 1: The Psychometric Hepatic Encephalopathy Score (PHES) is a “paper and pencil” screening test battery, designed for the diagnosis of minimal hepatic encephalopathy [20, 37]. This validated tool includes the following five tests: the line tracing test (LTT) with two scores for the same test: time and numbers of errors -motor speed and accuracy-, the serial dotting test (SDT) -motor speed-, the digit symbol test (DST) -associative learning; graphomotor speed, cognitive processing speed, visual perception, working memory-, the number connection test A (NCT-A) -psychomotor speed; visual scanning efficiency, sequencing, attention, concentration- and the number connection test B (NCT-B) -attention set shifting ability, psychomotor speed, visual scanning efficiency, sequencing, attention, concentration- all test are represented in Fig. 6 [38]. These tests examine motor speed and accuracy, visual perception, visuospatial orientation, visual construction, concentration, attention and to a lesser extent memory [22]. (DOCX 15 kb).

## Abbreviations

ACVRL 1: Activin receptor-like kinase type 1
BG: Basal ganglia
BG-MnIL: BG manganese induced lesions
CNS: Central nervous system
DMT1: Divalent Metal Transporter 1
DST: Digit symbol test
ENG: Endoglin
HHT: Hereditary hemorrhagic telangiectasia
HVMs: Hepatic vascular malformations
IDA: Iron deficiency anemia
LTT: Line tracing test
Mn: Manganese
MRI: Magnetic resonance images
MSCT: Triphasic multislice computarized tomography
NCT-A: Number connection test A
NCT-B: Number connection test B
PHES: Psychometric Hepatic Encephalopathy Score
SDT: Serial dotting test
TF: Transferrin
TGFβ: Transforming growth factor beta
