# Supplementary material for: Central nervous system manganese induced lesions and clinical consequences in patients with hereditary hemorrhagic telangiectasia
Source: Orphanet J Rare Dis. 2017 May 18;12:92. doi: 10.1186/s13023-017-0632-2 (PMC5437640; doi:10.1186/s13023-017-0632-2)
Supplement: Additional file 1: — The Psychometric Hepatic Encephalopathy Score (PHES) is a “paper and pencil” screening test battery, designed for the diagnosis of minimal hepatic encephalopathy [20, 37]. This validated tool includes the following five tests: the line tracing test (LTT) with two scores for the same test: time and numbers of errors -motor speed and accuracy-, the serial dotting test (SDT) -motor speed-, the digit symbol test (DST) -associative learning; graphomotor speed, cognitive processing speed, visual perception, working memory-, the number connection test A (NCT-A) -psychomotor speed; visual scanning efficiency, sequencing, attention, concentration- and the number connection test B (NCT-B) -attention set shifting ability, psychomotor speed, visual scanning efficiency, sequencing, attention, concentration- all test are represented in Fig. 6 [38]. These tests examine motor speed and accuracy, visual perception, visuospatial orientation, visual construction, concentration, attention and to a lesser extent memory [22]. (DOCX 15 kb). [file 13023_2017_632_MOESM1_ESM.docx]

Additional File1

The Psychometric Hepatic Encephalopathy Score (PHES) is a "paper and pencil" screening test battery, designed for the diagnosis of minimal hepatic encephalopathy. [^20^](#_ENREF_20)^,^[^37^](#_ENREF_37) This validated tool includes the following five tests: the line tracing test (LTT) with two scores for the same test: time and numbers of errors -motor speed and accuracy-, the serial dotting test (SDT) -motor speed-, the digit symbol test (DST) -associative learning; graphomotor speed, cognitive processing speed, visual perception, working memory-, the number connection test A (NCT-A) -psychomotor speed; visual scanning efficiency, sequencing, attention, concentration- and the number connection test B (NCT-B) -attention set shifting ability, psychomotor speed, visual scanning efficiency, sequencing, attention, concentration- all test are represented in figure 2.[^38^](#_ENREF_38) These tests examine motor speed and accuracy, visual perception, visuospatial orientation, visual construction, concentration, attention and to a lesser extent memory.[^22^](#_ENREF_22)
